# Runtime ML-DL Hybrid Inference Platform Based on Multiplexing Adaptive Space-Time Resolution for Fast Car Incident Prevention in Low-Power Embedded Systems

**DOI:** 10.3390/s22082998

**Published:** 2022-04-14

**Authors:** Sunghoon Hong, Daejin Park

**Affiliations:** School of Electronic and Electrical Engineering, Kyungpook National University, Daegu 41566, Korea; hopsison@knu.ac.kr

**Keywords:** deep learning, forward collision warning system, lightweight object detection, machine learning, vision processing

## Abstract

Forward vehicle detection is the key technique to preventing car incidents in front. Artificial intelligence (AI) techniques are used to more accurately detect vehicles, but AI-based vehicle detection takes a lot of processing time due to its high computational complexity. When there is a risk of collision with a vehicle in front, the slow detection speed of the vehicle may lead to an accident. To quickly detect a vehicle in real-time, a high-speed and lightweight vehicle detection technique with similar detection performance to that of an existing AI-based vehicle detection is required. In addition, to apply forward collision warning system (FCWS) technology to vehicles, it is important to provide high performance based on low-power embedded systems because the vehicle’s battery consumption must remain low. The vehicle detection algorithm occupies the most resources in FCWS. To reduce power consumption, it is important to reduce the computational complexity of an algorithm, that is, the amount of resources required to run it. This paper describes a method for fast, accurate forward vehicle detection using machine learning and deep learning. To detect a vehicle in consecutive images consistently, a Kalman filter is used to predict the bounding box based on the tracking algorithm and correct it based on the detection algorithm. As a result, its vehicle detection speed is about 25.85 times faster than deep-learning-based object detection is, and its detection accuracy is better than machine-learning-based object detection is.

## 1. Introduction

According to the World Health Organization (WHO), 1.3 million people die each year because of road traffic crashes [1]. Road crashes are very serious because many innocent people such as pedestrians, cyclists, and motorcyclists die in crashes every year. Most crashes are caused by driver inattention and poor judgment. One of the major challenges for autonomous driving is to ensure the safety of passengers and pedestrians by preventing vehicle collisions. Advanced driver assistance systems (ADAS) [2] help reduce traffic accidents caused by distracted driving. A forward collision warning system (FCWS) is one of the ADAS features that detects the risk that a vehicle installed with FCWS will collide with the vehicle in front of it and alerts the driver in the form of an audio notification, visual pop-up display, or other warning [3]. An FCWS can contribute significantly to reducing the number and severity of crashes by sending an alert to the driver about a possible impending collision if the vehicle gets too close to the vehicle in front of it.

In a 3-year study of more than 1900 vehicles, vehicles equipped with collision warning systems were involved in 78% fewer crashes. Providing the driver a 0.5-s warning of a rear-end collision can prevent as much as 60% of such crashes. If the warning time is given 1 s in advance, about 90% of rear-end collisions can be prevented [4]. To drive safely, a driver needs to estimate the distance to the vehicle in front, and it is very important to maintain a safe distance according to the vehicle speed. To calculate the distance to the vehicle in front, vehicle detection algorithms are necessary. In vehicle detection, powerful detection techniques such as deep learning cannot be applied to low-performance embedded systems. Machine-learning-based object detection is faster than deep learning is, but its detection accuracy is lower.

Many previous works have focused on a detection performance based on a single image. Therefore, in this paper, we propose a hybrid-based object detection method for faster, more accurate detection using machine learning and deep learning techniques in consecutive images. This module consists of three major steps. First, this module detects vehicles quickly and roughly in a single image using machine learning. Second, the bounding boxes of roughly detected vehicle candidate regions are more precisely corrected using deep learning. Lastly, object tracking is required to detect objects consistently in consecutive images. To prevent object tracking failure, a Kalman filter [5] is used to predict the bounding box based on the tracking result using template-matching [6] and to correct it based on the result detected in a single frame. The proposed algorithm, which outperformed the existing detection, works on both accuracies and running times in consecutive images.

The rest of the paper is structured as follows: Section 2 discusses the related research work in ADAS. Section 3 presents challenges in object detection. Section 4 introduces the hybrid-based object detection method, and Section 5 shows that the performance of the proposed architecture is much improved compared with the previous one. Finally, discussions and conclusion are mentioned in Section 6.

## 2. Related Research Work

As we know, the automobile industry is rapidly changing. The way vehicle works today is quite different than it was 10 years ago. The rapid change also requires safe driving to prevent traffic collisions, so, over time, the demand for ADAS in the automobile industry has increased and ADAS is widely used in many countries. According to the World Health Organization [7], more than 3500 people die every day on the roads, which amounts to nearly 1.3 million preventable deaths. Road traffic crashes have remained a major cause of death globally, even though every one of those deaths and injuries is preventable.

FCWS is an efficient approach to reduce the risk of collision into the vehicle in front. Extensive work has been done in this field, and this paper [8] is an attractive approach to fusing LiDAR and vision sensors to detect forward collision. 3D object classification [9] is an attractive approach to classify 3D objects using the LiDAR. In such a system, the LiDAR gives accurate range and range rate, while the vision detects the vehicle. However, this solution is expensive, and it is not easy to match the sensors well. On the other hand, a single vision system has a cost advantage because it does not require a separate sensor-matching operation and is simple to install. Vehicles can be detected in many ways using a single vision system. The method of detecting a vehicle using only edge information [10,11] results in many false detections.

To overcome this, a deep learning technique can detect vehicles using the You Only Look Once version 3 (YOLOv3) algorithm [12]. To compare the accuracy and speed of YOLOv3, YOLOv4, and YOLOv5, this paper [13] investigates the feasibility of using three state-of-the-art object detection algorithms. One-stage detectors such as YOLO [12,14,15], RetinaNet [16], and SSD [17,18] are generally more faster than two-stage detectors such as R-CNN [19] and variants [20,21,22,23]; however, they have low accuracy because of region proposal step is removed and both object localization and classification are done in the same step. Deep learning provides good detection performance, but the disadvantage is that it cannot be applied to a low-performance embedded system because a large amount of computation is required. Machine learning techniques such as vehicle detection using cascade classifiers [24] are faster than deep learning techniques are.

Detecting driver’s emotions [25] is another approach in ADAS that helps in FCWS. This model analyzes a driver’s emotions and generates a voice alert. Whenever it detects a positive driver emotion, it generates a moderate alarm for a period, and whenever it detects a negative emotion such as sadness, anger, anxiety, or disgust, it generates a highly elaborative alert to the driver and helps the driver understand the significance of safe driving. The driver’s emotions are detected through face recognition using a convolutional neural network (CNN) [26,27] for deep learning.

Fatigue detection [28] also works in ADAS, which alerts the driver to his or her condition by analyzing the driver’s mental state with the facial image. Driving while fatigued is a dangerous and common situation for drivers and represents a significant factor in fatal vehicle crashes. The facial-image-based fatigue detection system very accurately analyzes the drowsiness of the driver by extracting the regions of the eyes and mouth from a facial image. A local binary pattern [29] algorithm was used to extract features from the three regions, and the principal components were calculated using the principal component analysis [30] algorithm. This model works on machine learning using the support vector machine [31] algorithm.

## 3. Challenges in Object Detection

The field of computer vision has experienced significant progress in recent years due to advances in deep learning, especially CNNs [32]. The ultimate purpose of object detection is to find an unknown number of individual objects within an image, draw rectangular bounding boxes around them, and determine the class of each found object. However, not all of this progress is easy. Object detection presents many important challenges beyond what is required for image classification. As one of the most successful object detection families, YOLOv3 is fast and accurate. Here, we describe in detail two challenges facing YOLOv3 architecture.

### 3.1. Localization Problem

The localization problem occurs because YOLO [12,14,15] performs classification and localization at the same time for fast object detection. In the latest work, Redmon et al. [12] reported the following: “The performance drops significantly as the IOU threshold increases, indicating YOLOv3 struggles to get the boxes perfectly aligned with the object”. The last convolutional layer is ideal for classification because it is typically rich in terms of semantics. However, it is not ideal for localization because it is divided into grid units. Therefore, even if YOLO detects the same object in consecutive images, it makes larger localization errors compared to other two-stage detectors (R-CNN [19] and variants [20,21,22,23]) using region proposals where objects are likely to be located.

### 3.2. Speed Problem for Real-Time Detection

Object detection algorithms need to be incredibly fast to meet the typically real-time autonomous driving application or the real-time demands of video processing. One-stage detectors (YOLO [12,14,15], RetinaNet [16], and SSD [17,18]) perform object classification and localization simultaneously without a region proposal method. These algorithms detect objects faster because they have less complexity than two-stage detectors. YOLOv3 is faster than RetinaNet is and provides similar performance. It is important to strike a balance between detection speed and detection accuracy. YOLOv3 allows images of varying resolution such as 320 × 320, 416 × 416, and 608 × 608. In general, a high-resolution image is more accurate than a low-resolution image is, but processing time is slower. The problem with object detection design is that the choice must be made for the situation depending on whether speed or accuracy takes priority.

## 4. System Model and Methods

**Figure 1:**
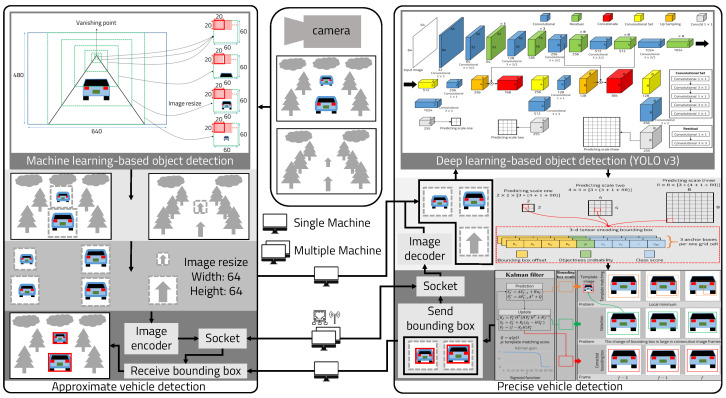
Lightweight object detection using a hybrid approach for low-power embedded systems.

We propose a lightweight object detection technique for detecting forward vehicles in low-power embedded systems. Our technique only applies to the FCWS process. We change neither the network architecture of deep learning nor the detection process. Figure 1 shows a lightweight object detection model using a hybrid approach for low-power embedded systems. The main idea of detecting forward vehicles quickly and accurately is to first detect them roughly and then detect them precisely within the bounding box of the detected vehicle. The proposed architecture is divided into two steps, one for approximate vehicle detection and one for precise vehicle detection. First, the approximate vehicle detection process receives images from the single camera sensor.

To detect a vehicle roughly from the input image, we use the Haar feature-based cascade classifier [24] algorithm based on machine learning. However, to detect only a vehicle in front, it is not necessary to detect vehicles for all regions in the input image. We use a vanishing-point-based cascade classifier [33] algorithm to detect vehicles only for meaningful regions in the input image. Second, to detect the vehicle accurately, we resize the detected vehicle to 64 × 64 and send it to the YOLOv3 framework. In the precise vehicle detection process, an image resized to 64 × 64 is detected precisely by the deep learning framework. Finally, the bounding box detected precisely through deep learning is corrected using the Kalman filter.

### 4.1. Machine-Learning-Based Object Detection

**Figure 2:**
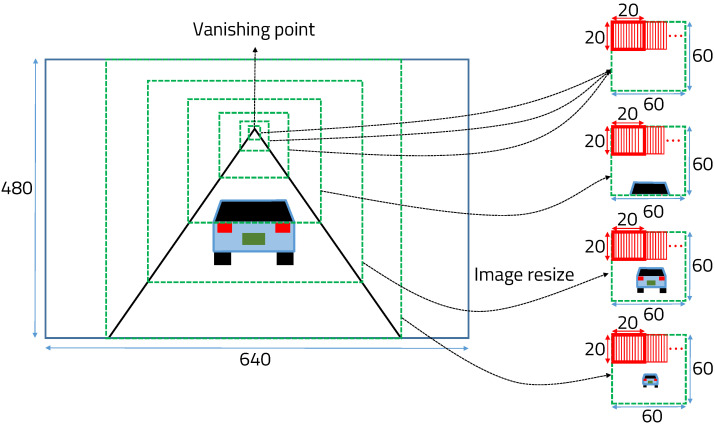
Vanishing-point-based cascade system.

The vehicle detection algorithm uses vanishing-point-based cascade classifiers with the Haar feature. To extract only meaningful features in vehicle detection, Adaboost [34] is used. Finally, the cascade function is trained using numerous positive images (images of vehicles) and negative images (images without vehicles) in a 20 × 20 window. The vanishing point helps to ensure that only meaningful regions are used for vehicle detection in the input image, as shown in Figure 2.

**Figure 3:**
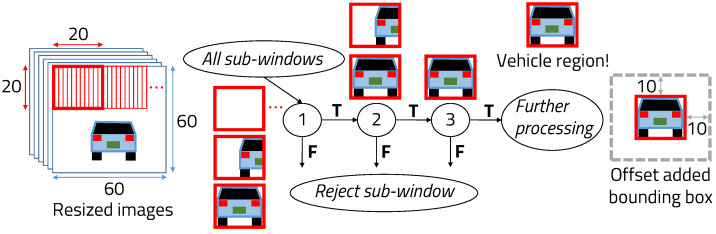
Cascade window sliding system.

The vanishing point is a point on the image plane where the two-dimensional perspective projections of mutually parallel lines in three-dimensional space meet at one point. In the case of a detected vehicle because the coordinates of the part touching the ground cannot exist above the vanishing point, they are calculated by dividing the windows into six steps. The cascade is used to detect vehicles in each region resized to 60 × 60 by dividing the windows in 6 steps, as shown in Figure 3. If a window fails in the first stage, it is discarded. If it passes, the second stage of features is applied and the process continues. The window that passes all stages is a vehicle region. However, the problem in the cascade function is to repeat sliding and increasing all windows in the input image. In fact, the window, such as the background is completely different from the vehicle, so even calculating it in the first stage of the cascade is inefficient. Because we use the cascade function only for resized meaningful regions where there is a vehicle using the vanishing point, the vehicle detection speed is much faster than applying cascade function on the original input image of 640 × 480. However, the machine learning technique encounters problems in that there are many false detections and large position errors for consecutively detected bounding boxes. To ensure that the vehicle is always in the detected bounding box, the offset value of the bounding box is added, as in the case of the gray bounding box.

### 4.2. Deep-Learning-Based Object Detection

**Figure 4:**
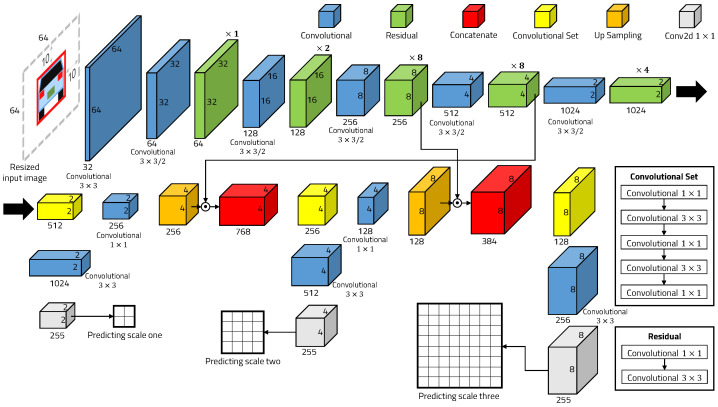
YOLOv3 framework.

YOLO is one of the most successful object detector families based on deep learning. We use the YOLOv3 framework to detect vehicles accurately. The deep learning technology has high computational complexity due to its many convolutional layers, so it is difficult to operate in real time in low-performance embedded systems. YOLOv3 has 53 convolutional layers, and it is called Darknet-53. One way to improve detection speed without modifying the existing network architecture is to reduce the size of the input image. However, if an image with an original size of 640 × 480 received from the camera is reduced to a smaller image of 64 × 64, then detection performance will be severely reduced because the object will also be smaller. To solve this problem, we use machine-learning-based object detection to quickly and roughly detect a vehicle and resize the image to 64 × 64 by adding an offset to the detected ROI. Since the image resized to 64 × 64 is entered as the input image of the YOLOv3 framework, it is possible to work in real-time and produce high-accuracy detection results, as shown in Figure 4. If two vehicles are close to each other, it can detect both vehicles, but only the vehicle in front of the current vehicle is detected using the vanishing point.

**Figure 5:**
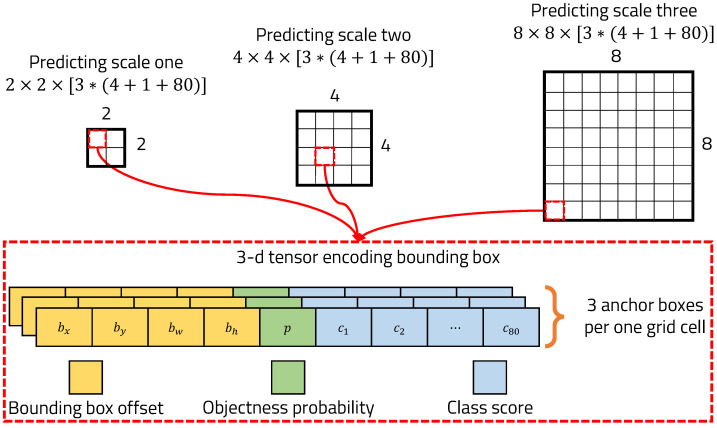
Predictions across scales.

YOLOv3 predicts bounding boxes at three different scales using dimension clusters as anchor boxes [35]. YOLOv3 extracts features from those scales using a similar concept to feature pyramid networks [36]. The last of YOLOv3’s network layers predicts a 3D tensor-encoding bounding box, objectness, and class score as shown in Figure 5. The tensor is N×N× [3 ∗ (4 + 1 + 80)] for the three bounding boxes, four bounding box offsets, one objectness probability, and 80 class scores with a COCO [37] data set.

**Figure 6:**
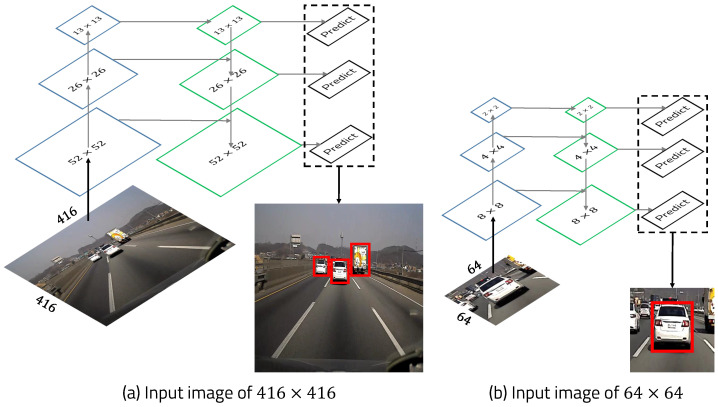
Feature pyramid network.

YOLOv3 performs prediction at three given scales by down sampling the input image dimensions of size 64 × 64 by 8, 4, and 2, respectively. Unlike the existing method that uses the input image of 416 × 416, we use the input image of 64 × 64 resized in a region quickly detected by machine learning, as shown in Figure 6.

For the input image of 416 × 416, the number of predicted bounding boxes is 10,647. On the other hand, for the input image of 64 × 64, the number of predicted bounding boxes is 252. This means that the proposed method predicts 42 times fewer bounding boxes than are predicted by the existing method. Because the number of predicted bounding boxes is reduced compared to the existing method, the detection speed is fast and the detection accuracy is similar.

### 4.3. Detected Bounding Box Correction

**Figure 7:**
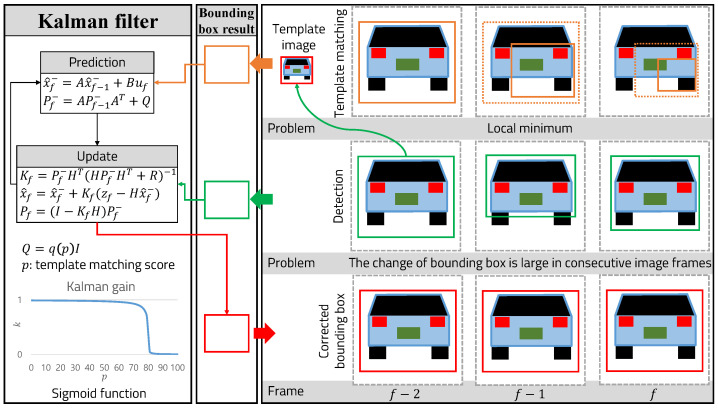
Object detection combining YOLOv3 and template-matching with a Kalman filter.

The problem with the deep-learning-based object detection algorithm is that it performs object detection by considering only a single frame. The position prediction of the bounding box detected for consecutive image frames may be inaccurate, and the probability of detection failure due to noise may increase. In this paper, we propose a detection and tracking algorithm for consecutive images by combining the YOLOv3 and the template-matching results with a Kalman filter to achieve higher vehicle detection accuracy, as shown in Figure 7. The Kalman filter is an efficient recursive filter that estimates the internal state of a linear dynamic system from a series of noisy measurements. Therefore, even if detection fails, object detection and tracking performance can be improved by reflecting object motion information between consecutive frames and removing noise.

We use a template-matching technique based on a template size of 20 × 20 for vehicle tracking. Template-matching is a technique in digital image processing for finding small parts of an input image that match a template image. In the case of an outdoor environment, a brightness change of the image is caused by sunlight or external factors. When a difference in the brightness occurs between the input image and the template image, it is necessary to normalize the image for reducing the brightness difference as shown in Equation (1):

(1) $$P\left(x,y\right)=1-\frac{\sum_{x^{{}^{\prime }},y^{{}^{\prime }}}{\left\{T\left(x^{{}^{\prime }},y^{{}^{\prime }}\right)-I\left(x+x^{{}^{\prime }},y+y^{{}^{\prime }}\right)\right\}}^{2}}{\sqrt{\sum_{x^{{}^{\prime }},y^{{}^{\prime }}}T{\left(x^{{}^{\prime }},y^{{}^{\prime }}\right)}^{2}\cdot \sum_{x^{{}^{\prime }},y^{{}^{\prime }}}I{\left(x+x^{{}^{\prime }},y+y^{{}^{\prime }}\right)}^{2}}}$$

*I* is the input image and *T* is the template image. (x,y) represents the coordinates of each pixel in the input image. $\left(x^{{}^{\prime }},y^{{}^{\prime }}\right)$ represent the coordinates of each pixel in the template image. *P* is the probability of the similarity to the template in coordinates of each pixel in the input image. The matching score has a value between 0 and 1; the position with the highest score is the best position. Since template-matching is not robust when image rotation change, if the matching score of the existing template image is less then 0.7, the image of the current region is updated with a new template image. In terms of the running speed of template-matching, considering all possible positions and sizes of the template with respect to the input image, the tracking speed decreases, so the proposed algorithm searches within a certain range based on the previously searched location to improve speed. Therefore, the template-matching-based tracking algorithm has problems such as tracking failure because it cannot recover to the ideal location because of local minimum problems, as shown with the orange bounding box. The green bounding box is the result of vehicle detection through the YOLOv3 framework, and its position error is largely due to image noise in consecutive frames.

To compensate for the problems of both algorithms, we use a Kalman filter. In the prediction step, the position of the bounding box is predicted using template-matching, and, in the update step, the predicted position is corrected using the result detected by YOLOv3 as a measurement value. If the template-matching result is incorrect, then YOLOv3 corrects it to prevent convergence to the local minimum, and the position detected by YOLOv3 has the effect of removing noise by template-matching. When modeling a Kalman filter, it is important to estimate *Q* (process noise covariance matrix) and *R* (measurement noise covariance matrix), and it is assumed that *A*, *B*, and *H* are identity matrices. *R* is the covariance matrix for the result detected by YOLOv3, and $M_{n}$ is a set of *m* vectors consisting of the *x* ($x_{center}$), *y* ($y_{center}$), *w* (width), and *h* (height) coordinates of the bounding box, as shown in Equation (2):

(2) $$Data:M_{n}=m_{1},\cdots ,m_{n}\left(m_{i}\in R^{4},m_{i}=\left[\begin{array}{c} x\\ y\\ w\\ h \end{array}\right]\right)$$

Given *n* pieces of data, *R* is calculated as shown in Equation (3):

(3) $$R=\frac{1}{n-1}\sum_{i=1}^{n}\left(m_{i}-{\tilde{m}}_{i}\right){\left(m_{i}-{\tilde{m}}_{i}\right)}^{T}$$

$\tilde{m}$ is the ideal position of the current frame tracked from the position of the bounding box of the previous frame using the result calculated from template-matching. The difference between the coordinates of the bounding box detected by YOLOv3 and the coordinates of the bounding box tracked by template-matching is given in Table 1.

The calculated *R* is as shown in Equation (4):

(4) $$R=\left[\begin{array}{cccc} 3.05 & -0.102 & 1.044 & -1.148\\ -0.102 & 6.928 & 0.463 & 3.542\\ 1.044 & 0.463 & 28.733 & -11.173\\ -1.148 & 3.542 & -11.173 & 34.418 \end{array}\right]$$

*Q* is the covariance matrix for the process, and it is estimated as shown in Equation (5):

(5) $$Q=\left[\begin{array}{cccc} q(p) & 0 & 0 & 0\\ 0 & q(p) & 0 & 0\\ 0 & 0 & q(p) & 0\\ 0 & 0 & 0 & q(p) \end{array}\right]$$

*Q* is composed of a diagonal matrix for the function q(p), and q(p) is calculated by combining two sigmoid functions as shown in Equation (6):

(6) $$q\left(p\right)=\frac{\alpha }{2}\left(\frac{\gamma (p-\beta )}{1+|\gamma (p-\beta )|}-1\right)\left(\frac{p-\beta }{1+e^{\gamma (p-\beta )}}-1\right)$$

A sigmoid function is a mathematical function having a characteristic S-shaped, or sigmoid curve. Sigmoid functions have a domain of all real numbers, and most such functions show a return value of the *y*-axis in the range 0 to 1. The value of *p* is a score calculated through template-matching, and has a value from 0 to 100. The α, β, and γ values were experimentally set to 42, 80, and 10, respectively, to minimize the error of the bounding box in consecutive images.

## 5. Experiments

In the test, the algorithm’s measurement speed was compared using the driving video previously stored in the LS1028A [38] board. LS1028A is a processor made by the NXP company, Eindhoven, Netherlands and is equipped with two powerful 64-bit Armv8 processors. It also features a built-in GPU, but because most low-power embedded systems lack this feature, it was not used in this paper.

LS1028A Specification:

- Model: Layerscape^®^ 1028A Applications Processor.
- CPU: 2-core Arm^®^v8 Cortex-A72 64-bit CPU 48 KB L1-I + 32 KB L1D + 1 MB L2.
- GPU: Vivante GC7000UltraLite.
- RAM: 4 GB 32-bit 1600 MTPS DDR4.
- Storage: 8 GB eMMC.
- Operating System: Linux operating system (an Ubuntu derived OS).

### 5.1. Execution Time

**Figure 8:**
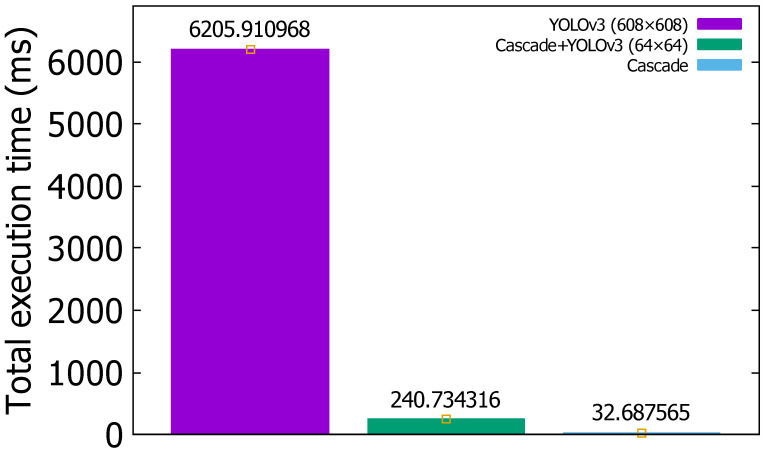
The total frame execution time of 1000 frames in YOLOv3, hybrid (cascade+YOLOv3), and cascade.

The total frame execution time of 1000 frames in the proposed hybrid object detection model is about 28.85 times faster than YOLOv3 is in the 608 × 608 input image, as shown in Figure 8. The cascade takes about 0.032 s and is about 7.5 times faster than the hybrid approach, but its detection accuracy is very low.

### 5.2. Kalman Filter Tuning

**Figure 9:**
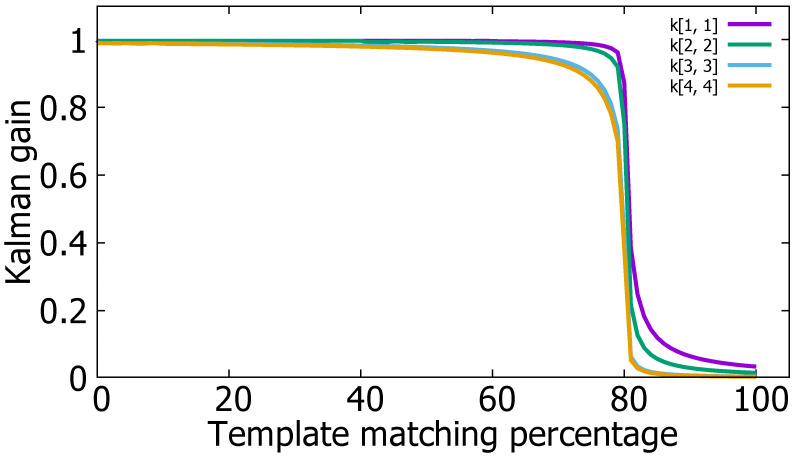
The change in the Kalman gain of the diagonal matrix according to the template-matching score.

The Kalman filter tuning problem is essentially a covariance estimation problem for process noise and measurement noise. The Kalman filter gain is computed based on the estimated covariances such as *Q* and *R* introduced in Section 4.3. The change in the Kalman gain of the diagonal matrix according to the template-matching score is shown in Figure 9.

R(1,1), R(2,2) are covariance of *x* and *y* centroid coordinates of the bounding box. R(3,3), R(4,4) are covariance of width and height of the bounding box. They are components of *R* (measurement covariance matrix) such as R(x,x), R(y,y), R(width,width), and R(height,height). When detecting vehicles with YOLOv3 in consecutive images, the width and height of the bounding box have a relatively larger error than *x* and *y* have. Therefore, when the template-matching percentage is greater than 80, the Kalman gain components such as K(3,3) and K(4,4) converge to almost 0. This means the width and height of the bounding box are predicted only through template-matching. However, even if the template-matching percentage exceeds 80, it can be seen that the Kalman-gain components such as K(1,1) and K(2,2) are not 0. This means the *x* and *y* centroid coordinates of the bounding box are slightly dependent on *x* and *y* centroid coordinates of the bounding box detected by the YOLOv3.

### 5.3. Detection Performance

**Figure 10:**
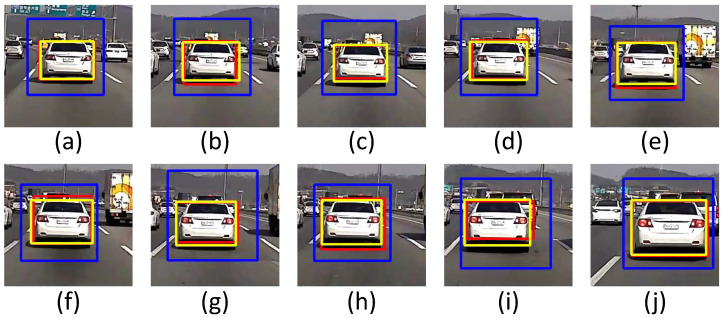
Vehicle detection results of the sedan: YOLOv3 (red), hybrid (yellow), and cascade (blue): (**a**) Current frame-90. (**b**) Current frame-80. (**c**) Current frame-70. (**d**) Current frame-60. (**e**) Current frame-50. (**f**) Current frame-40. (**g**) Current frame-30. (**h**) Current frame-20. (**i**) Current frame-10. **(j**) Current frame.

**Figure 11:**
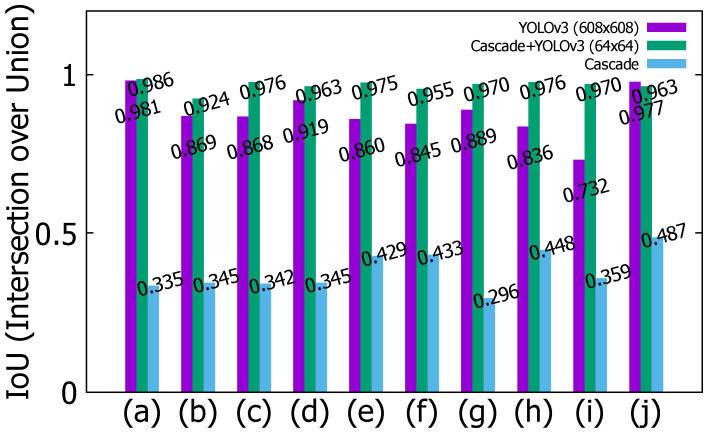
IoU results of the sedan for YOLOv3, hybrid (cascade+YOLOv3), and cascade: (**a**) Current frame-90. (**b**) Current frame-80. (**c**) Current frame-70. (**d**) Current frame-60. (**e**) Current frame-50. (**f**) Current frame-40. (**g**) Current frame-30. (**h**) Current frame-20. (**i**) Current frame-10. (**j**) Current frame.

The vehicle detection results of the sedan in YOLOv3, hybrid (cascade+YOLOv3), and cascade are shown in Figure 10. The blue bounding box is the result of cascade, red is the result of YOLOv3, and yellow is the result of the hybrid approach. In 10 consecutive frames from (a) to (j), the size and position of the blue bounding box are inaccurate. Red has a smaller position change than cascade has, but a small position error occurs. Yellow is more stable than cascade and YOLOv3 are. In 10 consecutive frames from (a) to (j), the measurement result of intersection over union (IoU) of the sedan is shown in Figure 11. IoU is an evaluation metric used to measure the accuracy of an object detector on a particular data set, and it is calculated as shown in Equation (7):

(7) $$\mathrm{IoU}=\frac{Area\;of\;Overlap}{Area\;of\;Union}$$

**Figure 12:**
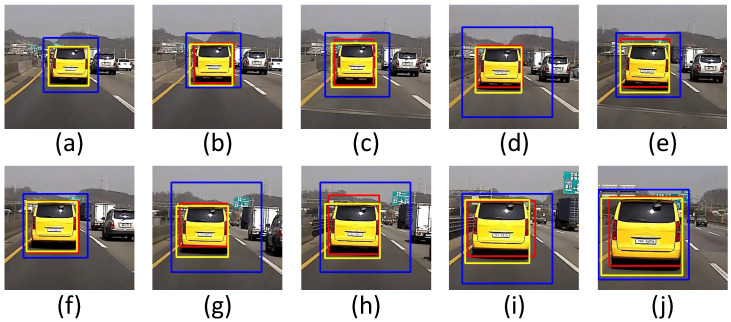
Vehicle detection results of the SUV: YOLOv3 (red), hybrid (yellow), and cascade (blue): (**a**) current frame-90. (**b**) current frame-80. **(c**) current frame-70. (**d**) current frame-60. (**e**) current frame-50. (**f**) current frame-40. (**g**) current frame-30. (**h**) current frame-20. (**i**) current frame-10. (**j**) current frame.

IoU has a value from 0 to 1, and the closer it is to 1, the higher the tracking accuracy becomes. It can be seen that the proposed method has a higher IoU value than other methods have. The vehicle detection results of the SUV in YOLOv3, hybrid (cascade + YOLOv3), and cascade are shown in Figure 12.

**Figure 13:**
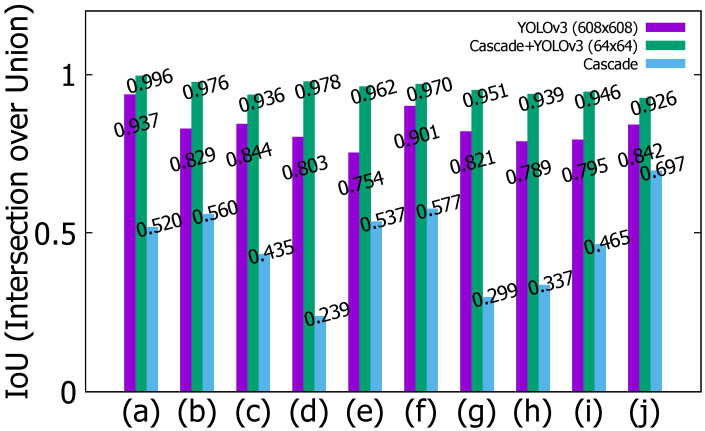
IoU results of the SUV for YOLOv3, hybrid (cascade+YOLOv3), and cascade: (**a**) current frame-90. (**b**) current frame-80. (**c**) current frame-70. (**d**) current frame-60. (**e**) current frame-50. (**f**) current frame-40. (**g**) current frame-30. (**h**) current frame-20. (**i**) current frame-10. (**j**) current frame.

In 10 consecutive frames from (a) to (j), the IoU measurement result of the SUV is shown in Figure 13. It shows that the bounding boxes of the detected vehicles are very inaccurate when using cascade: (d), (g), and (h). YOLOv3 also shows that the bounding boxes of the detected vehicles are not very accurate: (e), (h), and (i). In summary, the proposed method is immune to various interferences and works well in consecutive images.

## 6. Discussion and Conclusions

We presented a lightweight object detection method based on a hybrid approach using cascade and YOLOv3. We use the vanishing point to improve the approximate vehicle detection speed of machine learning. In deep-learning-based object detection, the detection speed is improved by about 25.85 times compared to the existing YOLOv3 because the image size is reduced. The vehicle detection accuracy is improved by correcting the results of template-matching and deep learning with a Kalman filter.

Future work will focus on further improving the performance of deep learning and lowering the power consumption of embedded systems using the state-of-the-art (SOTA) such as YOLOv4 and YOLOv5. We will also prove that the proposed method is efficient and good compared to other SOTA methods.

## Figures and Tables

**Table 1:** The difference of the bounding box coordinates for each frame.

Frame	1	2	3	⋯	498	499	500
$m_{x}-{\tilde{m}}_{x}$	1	1	2	⋯	−2	−2	−1
$m_{y}-{\tilde{m}}_{y}$	1	1	1	⋯	−2	4	−2
$m_{w}-{\tilde{m}}_{w}$	−3	−4	−2	⋯	2	7	2
$m_{h}-{\tilde{m}}_{h}$	6	7	−8	⋯	−14	−12	13

## Abbreviations

The following abbreviations are used in this manuscript:

ADAS	Advanced Driver Assistance Systems
FCWS	Forward Collision Warning System
WHO	World Health Organization
ML	Machine Learning
DL	Deep Learning
IoU	Intersection over Union
